# EFFECTS OF COMPANION ROBOTS ON DEPRESSION AND SOCIAL ISOLATION AMONG KOREAN AMERICAN OLDER ADULTS

**DOI:** 10.1093/geroni/igad104.1123

**Published:** 2023-12-21

**Authors:** Othelia Lee

**Affiliations:** University of North Carolina at Charlotte, Charlotte, North Carolina, United States

## Abstract

**Objectives:**

During the COVID-19, older immigrants in low resource areas who are already excluded from much of social life, had to distance themselves even further, deepening their social isolation. Lack of stimulation can be particularly detrimental, as it influences mood and increases loneliness. Selected for cost-effectiveness and interactive features, we introduced the Companion Robot (CR) system. Preliminary studies conducted in South Korea found statistically significant improvement in quality of sleep and cognition among low-income older patients living alone with multiple chronic conditions and cognitive impairment. Method. Thirty CRs were deployed to solo-living Korean American older adults. We followed up with them after 3 months. In this Stage 1A clinical trial, we tested feasibility, usability and acceptability of CRs, investigating which features of CRs will help improve health behaviors such as medication adherence and daily exercises. Robotic Social Attitude (RoSAS) are assessed with a generic 18-items questionnaire (Krägeloh et al., 2019).

**Results:**

The primary clinical outcomes measured at pre-and post-intervention were medication adherence and reduction of depression, social isolation and disabilities. The user-friendly doll-shaped design of CRs helped participants feel comfortable interacting with the CR on their own. Exposure to individualized CR-delivered content, including quizzes for brain training and inspirational messages, combined with consistent interaction with CR appeared to be beneficial for cognitive function.

**Discussion:**

Since very few participants had reported diagnosis of dementia, we need to screen for undetected dementia using the Modified Mini-Mental Status Exam. The next stage is to implement further cultural adaptation for the U.S. participants.

